# Minocycline reduces reactive gliosis in the rat model of hydrocephalus

**DOI:** 10.1186/1471-2202-13-148

**Published:** 2012-12-05

**Authors:** Hao Xu, Guowei Tan, Shaolin Zhang, Hongwei Zhu, Feng Liu, Caiquan Huang, Feifei Zhang, Zhanxiang Wang

**Affiliations:** 1 Department of Neurosurgery, First Affiliate Hospital of Xiamen University, Xiamen, Fujian Province, 361003, China; 2 School of Life Science, Xiamen University, Xiamen, Fujian Province, 361003, China; 3 Medical College of Xiamen University, Xiamen, Fujian Province, 361003, China

**Keywords:** Hydrocephalus, Gliosis, Astrocytosis, Microgliosis, Minocycline

## Abstract

**Background:**

Reactive gliosis had been implicated in injury and recovery patterns associated with hydrocephalus. Our aim is to determine the efficacy of minocycline, an antibiotic known for its anti-inflammatory properties, to reduce reactive gliosis and inhibit the development of hydrocephalus.

**Results:**

The ventricular dilatation were evaluated by MRI at 1-week post drugs treated, while GFAP and Iba-1were detected by RT-PCR, Immunohistochemistry and Western blot. The expression of GFAP and Iba-1 was significantly higher in hydrocephalic group compared with saline control group *(p < 0.05*). Minocycline treatment of hydrocephalic animals reduced the expression of GFAP and Iba-1 significantly (*p < 0.05*). Likewise, the severity of ventricular dilatation is lower in minocycline treated hydrocephalic animals compared with the no minocycline group (*p < 0.05*).

**Conclusion:**

Minocycline treatment is effective in reducing the gliosis and delaying the development of hydrocephalus with prospective to be the auxiliary therapeutic method of hydrocephalus.

## Background

Hydrocephalus is a common medical condition characterized by abnormalities in the secretion, circulation and resorption of cerebrospinal fluid (CSF), resulting in ventricular dilatation. Gliosis and neuroinflammation are prevalently associated with hydrocephalus, and reactive astrocytes and microglia have been found throughout the hydrocephalic brain, and followed by brain tissue fibrosis and function deterioration [1-7]. Previous studies have noted that reactive astrocytosis and microgliosis are commonly found in hydrocephalus rats with neonatal congenital [1,3,4,8] and acquired obstructive [2,6,8] hydrocephalus, with a perinatal onset followed by up to 5 weeks of postnatal ventriculomegaly. Astrocytosis has also been reported in juvenile and adult models of hydrocephalus [9,10].

As a second generation tetracycline-based molecule, minocycline is a potent inhibitor of microglia and astrocytes activation [11-15]. Because it is a highly lipophilic compound and can penetrate the brain-blood barrier easily, many studies used it for the treatment of central nervous system disease, and its role of reducing the brain injury after intracerebral hemorrhage has been reported [12,16,17].

We established hydrocephalus rats model by kaolin inducing and treated them with minocycline, then evaluated the ventricle enlargement by MRI. At the same time, the gene expression of GFAP (glial fibrillary acidic protein, for astrocytes) and Iba-1 (ionized calcium binding adaptor molecule, for microglia) were detected to explore the effectiveness of minocycline in reducing gliosis and the possibility of drug intervention as a new treatment strategies.

## Methods

### Animals

Adult male Sprague–Dawley rats (240–260 g) were purchased from Slac Laboratory Animal Co. Ltd. (Shanghai, CA). Food and water were provided ad libitum. Animals were housed in a temperature- and humidity-controlled environment with a 12-h light/dark cycle. The rats were randomly divided into 2 groups: experimental group (n=20) with kaolin injections, and control group (n=10) with saline injections. Then the model group were randomly subdivided into the untreated hydrocephalic group (n=9) and the minocycline-treated hydrocephalic group (n=9) at 2-week post-operation. Experimental procedures were performed according to the guidelines of the Animal Experiment Ethics Committee of Xiamen University.

### Surgical induction

All the intervener and evaluator were blinded. The rats were anaesthetized by intraperitoneal injection of 10% chloral hydrate (0.4 ml/100 g), and were placed in a stereotaxic frame. The injection coordinates, which were measured from the bregma to the lateral cerebral ventricles, were 0.8 mm posterior, 1.6 mm lateral and 3.7 mm deep. A 30-μl sterile suspension of 3% kaolin (ultrasonic emulsification about 15 minutes) was injected slowly into the lateral cerebral ventricles (depth: 3.7 mm) at a rate of approximately 10 μl/min. The skin incision was sutured, and the animal was replaced in its cage and was monitored daily for the duration of the experiment. Sham control rats underwent the same procedure but received sterile saline injection instead of kaolin [18].

Due to the small size of the young rats used in this study (250 g for adults) intravenous injection was not a practical method of minocycline delivery. Therefore, intraperitoneal injections were used instead [7,19]. This type of administration has been shown to provide adequate delivery of minocycline to the brain across the blood brain barrier as assessed by both serum and CSF levels [20]. Minocycline treated animals received minocycline HCl (Sigma Chemicals, St. Louis, USA) dissolved in 5% sucrose at a concentration of 10 mg/ml at a dose of 45 mg/kg/day for 7 days, beginning at post-injection day 15 and ending at day 21 with the termination of the experiment. Injection sites were rotated between the four abdominal quadrants.

### Magnetic resonance imaging and ventricular measurement

Magnetic resonance imaging (MRI) was performed on all animals 14 days post-injection. The animals were anesthetized with chloralic hydras and all scans were performed on a 7.0-Tesla horizontal-bore small animal MRI scanner (BrukerBiospec, Billerica, MA, USA) abiding by the protocol described previously. Evan's ratios [21] and ventricular volumes were measured from the coronal MRI scans of the saline control and hydrocephalic brains.

Ventricular volumes (lateral and 3rd ventricles) were calculated according to T2 images, starting from the center of the cerebral aqueduct up to the anterior-most portion of the lateral ventricles. The volumetric calculations were semiautomated(by NIH Image)as follows: an appropriate intensity threshold was first chosen to exclude background tissue and to highlight the bright ventricles. This was followed by careful inspection of each image, and manual tracing was used to correct any area of the ventricle, which had been incorrectly deleted, or to delete nonventricular regions that had been incorrectly included. This process resulted in a binary mask of ventricular pixels, which multiplied by the volume of each pixel and summed over all slices produced the net ventricular volume in milliliters. The Evan’s ratio was taken at the level of the Foramen of Monroe as the maximum width of both lateral ventricles divided by the maximum width of the brain at this level.

### Sacrifice and tissue sample collection

**Figure 1:**
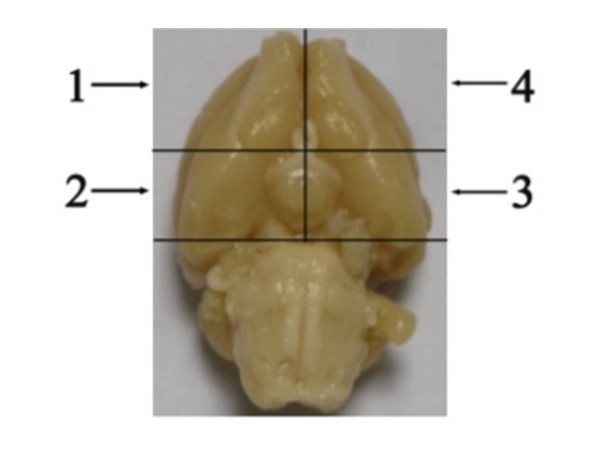
**The relative locations of a rat brain used for various assays in this study.** The first part for pathological study the second for immunohistochemical study; the third for Western blotting and the fourth for qRT-PCR analysis

On post-treated day 8 following the MRI, each group of rats were anesthetized with 10% chloral hydrate (0.4 ml/100 g, i. p.) and perfused with 0.9% saline by left atrial perfusion. For histological analysis, the brains were removed from the skull. Each brain was divided into four parts between optic chiasma and cerebral peduncle [22,23]. The first and second parts of the brain were fixed in 10% formalin for 24 h at 4°C and then embedded in paraffin for histological and immunohistochemical studies respectively. And the third and fourth parts were stored at -80°C for western blot and qRT-PCR respectively (Figure 1). 

### RT-PCR

Total RNA was extracted by Trizol reagent (Invitrogen, USA) according to the manufacturer's instructions. Reverse transcription and qRT-PCR were performed using commercially available reagents (TaKaRa, Japan) with the StepOne Real-Time PCR System (ABI, UK). Sybr Green I was used to detect amplification and β-actin was used as a normalizing control. Forward and reverse primers were as follows (Table 1):

**Table 1:** Primer sequences used for qRT-PCR

**Primer name**	**Sequences**
β-actin	Forward 5’-CAACCTTCTTGCAGCTCCTC-3^′^
	Reverse 5’-CCCTCATAGATGGGCACAGT-3^′^
GFAP	Forward 5’-GACCGCTTTGCTAGCTACATCG-3^′^
	Reverse 5’-GGTTTCATCTTGGAGCTTCTGC-3^′^
AIF-1	Forward 5’-GAATTCACCATGAGCCAAAC 3^′^
	Reverse 5’-GGATCCTTAGGGCAACTCAG 3^′^

The RT-PCR consisted of two programs. The first one was complementary cDNA synthesis: one cycle at 50°C for 30 min and one cycle at 94°C for 2 min. The other one was second-strand cDNA synthesis and the PCR consisted of 45 cycles at 94°C for 30s, 58°C for 30s, and 72°C for 20s, followed by a final extension step at 72°C for 10 min. Calculations were performed by the CP method. All samples were assayed in triplicates.

### Immunohistochemistry

Paraffin-embedded brains of optic chiasma which is of the lateral ventricle level were sectioned coronally at a thickness of 5 μm, and were immunohistochemically stained for detecting the expression of GFAP and Iba-1 with 2-step EliViaion+ kit (Maixin. Bio, CA) according to the manufacturer's instructions. In brief, after dewaxing and rinsing with PBS, for antigen retrieval, sections were microwave-treated in a steamer for 20 min with 10 mmol citrate buffer (pH 6.0) preheated to 90-100°C, followed by rinsing again and incubation in 3% H_2_O_2_ for 10 min. Tissue sections were then blocked with blocking serum for 20 min. The tissue sections were incubated with monoclonal antibody against GFAP (1:1000) and with polyclonal antibody against Iba-1 (1:200) (abcom Biotechnology, USA) respectively, overnight at 4°C in a moist chamber. Then, the slides were incubated for 30 min at 37°C with Elivison+, peroxidase, rabbit (for GFAP and Iba-1) and subsequently reacted with 3,3-diaminobenzidine (DAB) mixed solution (Maixin. Bio, CA)for 2–3 min as a chromogen substrate. The nucleus was counterstained by Meyer hematoxylin. Then, the slides were dehydrated and mounted. Negative controls were incubated without primary antibodies.

### Western blotting

Anesthetized animals were decapitated, and the brain was promptly removed and dissected on ice. Samples were placed in sterile tubes and immediately submerged in liquid nitrogen, and then transferred and stored in −80°C until use. Tissue from Sham Control group(n=10), Hydrocephalic with minocycline group(n=9) and Hydrocephalic no minocycline group(n=9) was placed in separate vials containing RIPA buffer(Pierce 89900) with a protease inhibitor tablet (Roche-Complete Mini 4°C)and sonicated. After centrifugation at 13,000 rpm for 10 min at 4°C, the supernatant was aliquoted and frozen with a sample from each animal retained for protein quantification. Protein quantification was performed using BCA Protein Assay Kit (Thermo Scientific, Pittsburg PA.). Absolute protein content was calculated using Lowry method [24].

Proteins from the control and hydrocephalic parietal cortices were obtained at appropriate working concentrations by mixed with an equal volume of Laemmli buffer with -mercaptoethanol and heated at 90°C for 5 min, loaded into each lane, and electrophoresed in a 10% SDS-polyacrylamide gel in 1×Tris/Glycine/SDS buffer at 150 V for 1.5 h at room temperature. Separated proteins were electrophoretically transferred to a PVDF membrane using 1×Tris/Glycine buffer for 30 min at 100 V and 4°C. The membrane was washed in Tris-buffered saline +Tween (TBS-t) and blocked in 5% milk for 1 h at RT. Primary antibody for GFAP (abcom, Cambridge US.) and Iba-1(abcom, Cambridge US.) the reference protein GAPDH(abcom, Cambridge US.) were applied at a dilution of 1:1,000 for 2 h at RT followed by 3 washes in TBS-t. Secondary antibody, peroxidase-conjugate anti-rat IgG, was applied at a dilution of 1:1,000 for 1 h followed by 3 washes in TBS-t. Finally, ECL reagent was added to the membrane, and the membrane was exposed on film. Western blots were quantified by densitometry readings from the appropriate bands (GFAP 50 kDa and Iba-1 17 kDa). To accurately quantify the bands, the relative intensities of GFAP and Iba-1 were divided by the relative intensity of our loading control GAPDH (37 kDa).

### Data analysis

Data were generally expressed as mean ± standard error (SE) values. Groups of data were compared by a one-way ANOVA. Two comparisons were made - between the saline control and hydrocephalic animals; and between hydrocephalic with minocycline and hydrocephalic without minocycline groups. A value of P<0.05 was considered to be statistically significant.

## Results

### Behavior, mortality and general changes

Rats were watched and weighed daily. Two rats in the hydrocephalic group died within 3 days post-operation. All rats with kaolin injection into the lateral ventricle successfully developed moderate to severe hydrocephalus according to MRI examination. Most rats of hydrocephalic groups exhibited irritability, increased urine, coughing, hunched back, hind legs weakness and nasal and/or orbital secretions of blood and clear fluid after 1 day post-operation. These signs disappeared spontaneously within a few days. Weight growth of hydrocephalus animals was significantly slower than that of the sham control group. Besides, there is no significant difference between Hydrocephalic with minocycline rats and Hydrocephalic no minocycline group.

### MRI

**Figure 2:**
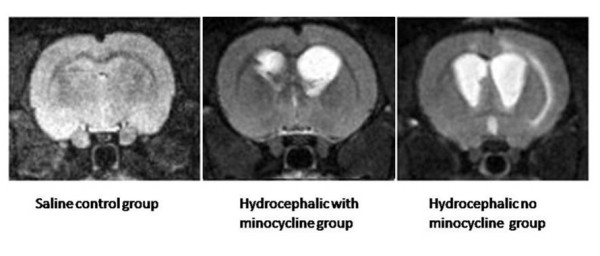
**Coronal T**_**2**_**-weighted MR images of controls and rats with hydrocephalus are shown**

Animals with saline injections into the lateral ventricle did not develop hydrocephalus; however, all rats with kaolin injections developed hydrocephalus. In these animals, MRI confirmed ventriculomegaly (*P*<*0.05*). The lateral ventricles enlarged equally, which confirmed that the obstruction was between spinal and cortical subarachnoid space (in traditional concept it was classified of communicating hydrocephalus) [25]. Severely hydrocephalic brains exhibited dramatic expansion of the lateral ventricles, as well as a parietal cortex that was substantially thinned and edematous. The third ventricle, cerebral aqueduct, and fourth ventricle were enlarged as well, but not to the same degree as the lateral ventricles (Figure 2). Before drug treatment, the MRI results showed no difference on the Ventricular volumes and Evan’s ratio between the two groups. The ventricular volumes of both the minocycline group and the no minocycline group were enlarged after drug treatment period. Yet, the Ventricular volumes and Evan’s ratio of minocycline group is smaller (*P*<*0.05*) compared to the no minocycline group (Table 2). 

**Table 2:** Mean ventricular volumes and Evan’s rate

**Experimental model**	**Saline Control group**	**Before treated**		**After treated**	
		**Hydrocephalic with minocycline group**	**Hydrocephalic no minocycline group**	**Hydrocephalic with minocycline group**	**Hydrocephalic no minocycline group**
	**(n=10)**	**(n=9)**	**(n=9)**	**(n=9)**	**(n=9)**
Ventricular volumes(mm^3^)	16±8	49±10	57±12	98±18	140±21
Evan’s ratio	0.29±0.04	0.33±0.03	0.34±0.04	0.42±0.05	0.56±0.09
Body weight(g)	381	311	326	315	349

### RT-PCR

**Figure 3:**
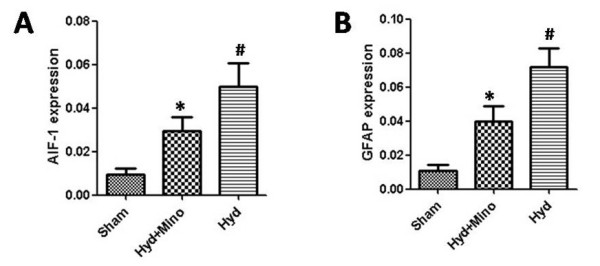
**Mean ranking score for GFAP(A) and AIF-1(B) expression in brain tissue for sham Control group(n=10), Hydrocephalic with minocycline group(n=9) and Hydrocephalic no minocycline group(n=9).** The result were expressed GFAP/β-actin or Iba-1/β-actin. The Hydrocephalic no minocycline rats expressed levels increased(*P<0.01*) of GFAP protein and AIF-1 when compared with Sham control animals(#). The GFAP and AIF-1 expression of Hydrocephalic with minocycline group are lower(*P<0.05*) than that of no minocycline group(*)

RT-PCR (Figure 3) revealed that the mRNA expression of the GFAP and AIF-1 significantly (*P*<*0.05*) elevated in hydrocephalic brain. Meanwhile, the level evaluation is more obvious (*P*<*0.05*) in hydrocephalic no minocycline group than that of the minocycline group and it was revealed by correlation analysis that the increase was positively correlated with the severity of ventricular dilatation.

### Immunohistochemistry

**Figure 4:**
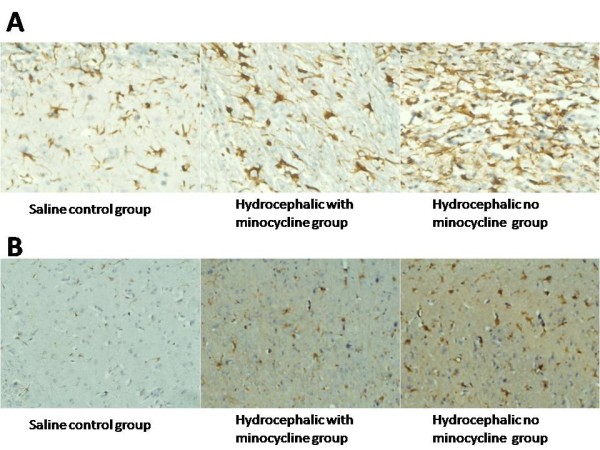
Representive images of GFAP(A) and Iba-1(B) of the preventricular tissue (white matter dorsal to frontal horn of lateral ventricle)

GFAP and Iba-1 immunostaining (Figure 4) revealed both resting and reactive glial cells in preventricular tissue and parietal cortex. The parietal cortex in saline control animals contained predominantly resting astrocytes and microglial cells, which are both characterized by a lightly stained cell body and thin processes. In the hydrocephalic no minocycline group, highly reactive astrocytes, which had the typical appearance of dark enlarged cell bodies and thick processes, were found throughout the cortex. Likewise, densely-packed reactive microglial cells were also frequently found in preventricular area and cortex of these animals. In the hydrocephalic with minocycline group, the typical appearance of dark enlarged cell bodies was relatively fewer than in the no minocycline group.

### Western blot analysis

**Figure 5:**
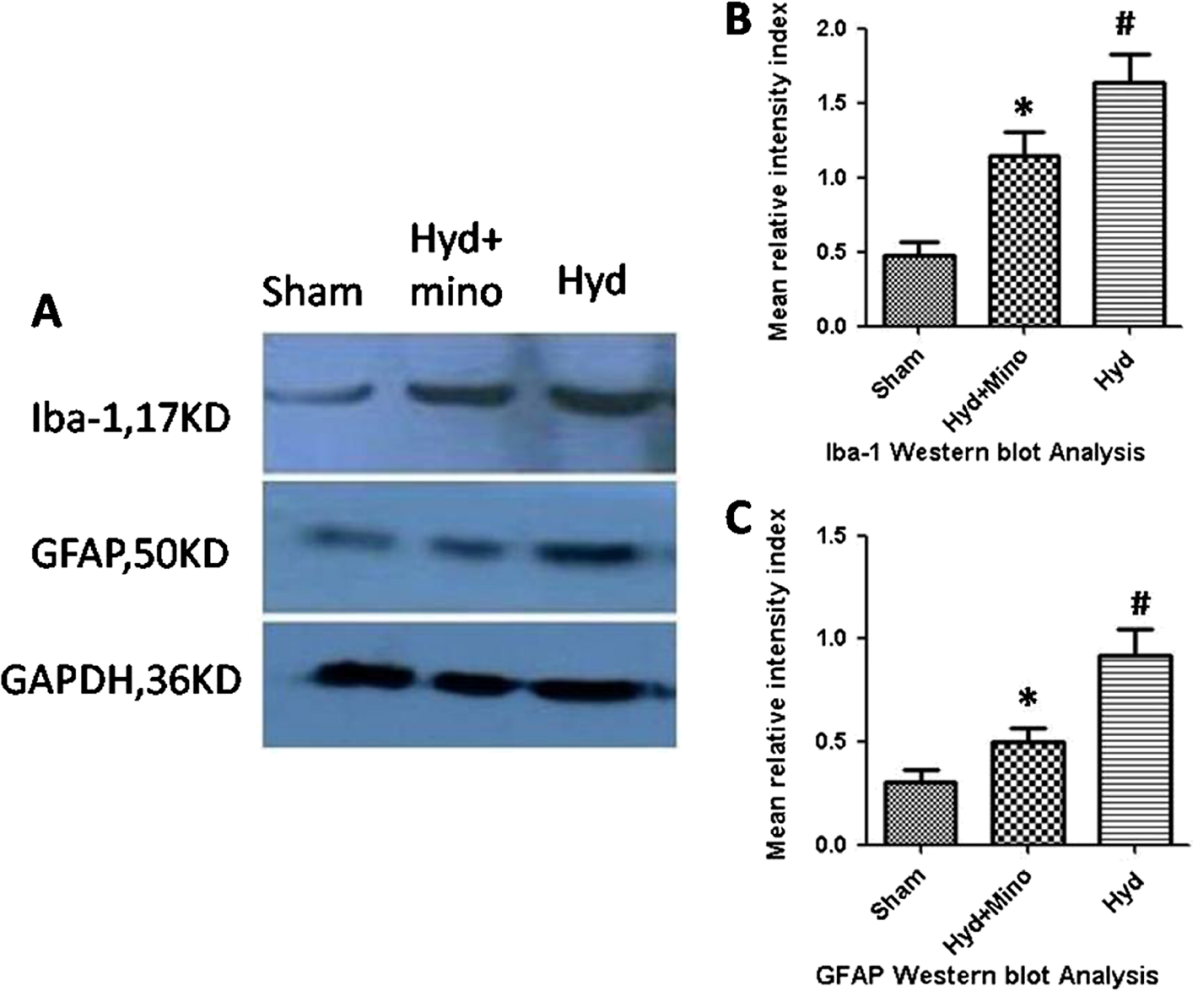
**Proteins were processed for Western blot to assess levels of GFAP and Iba-1(A).** Mean ranking score of GFAP(**B**) and Iba-1(**C**) in brain tissue for Sham Control group(n=10), Hydrocephalic with minocycline group(n=9) and Hydrocephalic no minocycline group(n=9) were analysed. The Hydrocephalic no minocycline group expressed levels significantly(*P<0.01*) increased of GFAP and Iba-1 protein when compared with Sham control animals (#). The GFAP and Iba-1 expression of Hydrocephalic with minocycline group are lower(*P<0.05*) than that of no minocycline group(*)

GFAP protein and Iba-1 expression increased significantly (*P*<*0.01*) in the hydrocephalic groups compared to the saline control group. In hydrocephalic with minocycline group, GFAP protein and Iba-1 expression are lower than hydrocephalic no minocycline group (*P*<*0.05*). It revealed by correlation analysis that the decrease was positively correlated with the severity of ventricular dilatation (Figure 5).

## Discussion

In traditional concept, hydrocephalus was divided into obstructive and communicating. And when the obstruction occurs between spinal and cortical subarachnoid space, it was classified as communicating hydrocephalus. Recently, a consensus on the classification of hydrocephalus was reached [26], kaolin deposition in subarachnoid space is also a resistant elements within the CSF pathways, so the model cannot be classified into communicating hydrocephalus.

In the present study, our RT-PCR, Immunohistochemistry and Western blot all confirm that minocycline can reduce the expression of GFAP and Iba-1 in hydrocephalic rats. Although it cannot revise the development of hydrocephalus, minocycline can delay its progress. The mechanism might operate through inhibition of astrogliosis and glial scar formation thereby decreasing the damage to periventricular white matter. GFAP is an intermediate filament specifically seen in astrocytes. As to functions of reactive astrocytes, it has been reported that reactive astrogliosis and glial scar formation play crucial roles in regulating CNS inflammation [27]. Glial scar formation could play a major role in causing the problem that chronically plague hydrocephalic children. It has been suggested by many investigators [28] that GFAP-labeled reactive astrocytes were present in different hydrocephalic animal models. Clinically, increased levels of GFAP protein have been found in the CSF of patients with normal pressure hydrocephalus, and patients who developed secondary hydrocephalus due to subarachnoid hemorrhage [29-31]. At the same time, the possibility of using GFAP protein levels as a diagnostic tool for hydrocephalus is currently being explored [32,33].

Astrocytes, in nature, seem to perform a regulatory function in the processes of microglial differentiation and activation in response to inflammation, considering their role in the antigen-presenting process. Extracellular glutamate levels increased and astrocytes are stimulated in brain injury, thus producing calcium wave propagations and releasing nucleosides and nucleotides which in turn enable a stimulation of proliferation of microglia [34-36], with a wide production of molecules, including cytokines. The cytokines interleukin 1β (IL-1β), interferon-γ (IFN-γ), and tumor necrosis factor (TNF-α) are important for microglial activation since they can induce proliferation and functional changes in microglial cells. However, as they also act upon astrocytes, they can create cascade effects contributing to both perpetuation of inflammation by a vicious circle and affecting neuronal function. Coupled with microglia, astrocytes also release trophic factors such as basic fibroblast growth factor, nerve growth factor (NGF), ciliaryneurotrophic factor, and S100β, thus promoting neuroplasticity and rebuilding of the nervous system after injury. The release of trophic factors is possibly mediated by ATP, which triggers a form of calcium communication between astrocytes and microglia in tissue repair pathways by stimulating purinergic receptors [37,38].

Microglia are cells within the brain which are activated in response to injury. Depending upon specific conditions, they can have neurotrophic or neurotoxic actions. In normal brain, microglia are in a quiescent state; However in the event of injury, they become highly phagocytic and they are involved in clearing debris from the damaged site. Activated microglia are found associated with ischemic and hemorrhagic brain injury, including intracerebral hemorrhage. Activated microglia upregulate a variety of surface receptors, including the major histocompatibility complex (MHC) and complement receptors [39], and release a wide variety of proinflammatory mediators such as cytokines, chemokine, proteolytic enzymes, reactive oxygen species, and complement protein [40]. Activated microglia are found associated with ischemic and hemorrhagic brain injury, including intracerebral hemorrhage. Our previous study also confirmed that the degrees of astrocytpsis and microgliosis are positively related to the development of hydrocephalus and indicated a detrimental role of glial cells in hydrocephalus despite of the protective role of reactive gliosis [23].

Minocycline, a second generation tetracycline-based molecule, is a highly lipophilic compound and penetrates the brain–blood barrier easily [16,41]. Minocycline has neuroprotective effect such as anti-oxidation, anti-inflammatory, anti-apoptosis [42-44], and its protection role in ischemiare perfusion injury and neuropathic pain has been reported [45]. An in vitro study showed that minocycline reduced excitotoxicity in primary neuronal culture by preventing excitotoxin-induced microglial proliferation [11]. It also inhibits macrophage/microglia activation after intracerebral hemorrhage in the rat [17,46]. Another study suggested that minocycline reduce the late histopathological consequences by restoring sAPPα levels [47]. Overall, these data suggested that minocycline treatment is effective in reducing the gliosis and the severity of hydrocephalus with prospective to be the auxiliary therapeutic method of hydrocephalus.

## Conclusion

To hydrocephalic patients, surgery can only release symptoms while etiological treatment intend to be more promising therapeutic strategy. As multiple factors contribute to the development of hydrocephalus, despite of the experiment confirmed the effectiveness of minocycline to some degree, its underling mechanism is yet unclear and needs further exploration.

## Competing interests

The authors declare that they have no competing interests.

## Authors’ contributions

HX and ZW conceived and designed the experiments. GT participated in the study design, carried out all of the experiments.SZ and HZ conducted the animal experiments. LF conducted the RT-PCR experiments. CH conducted the Western blotting experiments. FZ conducted the statistical analyses. All authors read and approved the final manuscript.
